# Interspecific variation of calls in clownfishes: degree of similarity in closely related species

**DOI:** 10.1186/1471-2148-11-365

**Published:** 2011-12-19

**Authors:** Orphal Colleye, Pierre Vandewalle, Déborah Lanterbecq, David Lecchini, Eric Parmentier

**Affiliations:** 1 Laboratoire de Morphologie Fonctionnelle et Evolutive, Université de Liège, Allée de la Chimie 3, 4000 Liège, Belgium; 2 Laboratoire de Biologie Marine, Université de Mons-Hainaut, Avenue du Champ de Mars 6, 7000 Mons, Belgium; 3 Centre de Recherche Insulaire et Observatoire de l'Environnement (CRIOBE), USR 3278 CNRS-EPHE, 1013 Papetoia Moorea, French Polynesia

**Keywords:** Pomacentridae, clownfish, evolution, biogeography, sound production, sympatry, hierarchy, size, frequency

## Abstract

**Background:**

Clownfishes are colorful coral reef fishes living in groups in association with sea anemones throughout the Indo-Pacific Ocean. Within their small societies, size hierarchy determines which fish have access to reproduction. These fishes are also prolific callers whose aggressive sounds seem to play an important role in the social hierarchy. Agonistic interactions being involved in daily behaviour suggest how acoustic communication might play an important role in clownfish group. Sounds were recorded and compared in fourteen clownfish species (some of which have never been recorded before) to evaluate the potential role of acoustic communication as an evolutionary driving force.

**Results:**

Surprisingly, the relationship between fish size and both dominant frequency and pulse duration is not only species-specific; all the specimens of the 14 species are situated on exactly the same slope, which means the size of any *Amphiprion *can be predicted by both acoustic features. The number of pulses broadly overlaps among species, whereas the pulse period displays the most variation even if it shows overlap among sympatric species. Sound comparisons between three species (*A. akallopisos, A. ocellaris *and *A. frenatus*) having different types of teeth and body shape do not show differences neither in the acoustic waveform nor in the power spectrum.

**Conclusion:**

Significant overlap in acoustic features demonstrates that the sound-producing mechanism is highly conservative among species. Differences in the calls of some species are due to size dimorphism and the sound variation might be in this case a by-product. This morphological constraint does not permit a consideration of acoustic communication as the main driving force in the diversification of clownfishes. Moreover, calls are not produced to find mate and consequently are less subject to variations due to partner preference, which restricts the constraints of diversification. Calls are produced to reach and defend the competition to mate access. However, differences in the pulse period between cohabiting species show that, in some case, sounds can help to differentiate the species, to prevent competition between cohabiting species and to promote the diversification of taxa.

## Background

Acoustic communication provides a good model for studying the evolution of behaviour. Generally speaking, acoustic signals used in mate choice and mate recognition can play a role in speciation for sound producing taxa such as anurans [1,2], insects [3,4] and birds [5,6]. Variation in acoustic signals can act as pre-zygotic isolating mechanisms [7-9], the receivers being the selective force on the evolution of the signal [10]. The acoustic signal is typically composed of both temporal and spectral components, which may evolve at different rates [11]. Examining these patterns of acoustic variation among species may be useful for testing the evolutionary history of the characters [12,13].

Among the sound-producing fish, the coral reef damselfishes (Pomacentridae) are one of the most intensely studied families with sound production being documented for more than 20 species, belonging to seven different genera [14-16]. Within this large and diverse fish family, clownfishes are colorful coral reef fishes well known for their mutualistic relationship with tropical sea anemones that host them [17]. Recent phylogenetic investigations using both morphological and molecular characters supported the monophyly of the clownfishes belonging to the genera *Amphiprion *and *Premnas *[18-20]. More specifically, on the basis of 23 (out of 28) species and three mitochondrial genes, Santini and Polacco [20] proposed a first hypothesis concerning the lifestyle and origin of the ancestral clownfish. They suggested it was a slender-bodied animal with a rounded caudal fin. However, their interpretation of the evolutionary history of this group still remains poorly explained.

In clownfishes, groups are composed of a breeding pair and between zero to four non-breeders, depending on species and size of host [21,22]. Within each group, the sex is controlled socially and there is a size-based dominance hierarchy: the breeding female is the largest individual, the breeding male is the second largest and the non-breeders get progressively smaller as the hierarchy descends [22,23]. The size hierarchy forms a queue to attain dominant status; individuals only ascend in rank when a higher rank individual disappears, and the smallest fish in the group is always the most recent recruit [21,24]. Clownfishes are prolific callers, producing sounds during interactions among group members [16,25]. In such a system, sounds are not used for mate attraction. However, acoustic signals might confer a higher probability of attaining breeding status. Dominant frequency and pulse duration of the calls being morphologically determined signals related to fish size [26,27], sounds seem to be important for living in social group because the hierarchy determines which fish can have access to reproduction [21].

Agonistic interactions are involved in daily behavior [22] and sounds are known to be associated with them [17,26,28], suggesting how important acoustic communication is in clownfish group. So, the question arises as to whether this behaviour is also important in speciation process. To find out, the first step was to compare the calls of closely related clownfish species in order to evaluate the variation in call characters [see [7]]. *Amphiprion *species produce the same kind of broadband-pulsed sounds during agonistic interactions [17,27,29], meaning the differences should not be at the level of the biomechanics. Although sounds are all produced by snapping jaws [30], other characteristics could display variation among species. Moreover, dominant frequency and pulse duration are known to be size-related acoustic signals in clownfishes [26,27]. We predict that if these characters were important in the taxon diversification, the different species would have evolved different relationships between dominant frequency and fish size, and between pulse duration and fish size. Overlap in other characters such as pulse period or number of pulses in a call would not be observable in case of diversifying character of the taxa. This study analysed the sounds of 14 different clownfish species. The aim was to test the hypothesis that acoustic features can help to evaluate the potential role of acoustic communication as a driving force in the evolution of clownfishes.

## Results

### Interspecific differences in sounds

**Figure 1:**
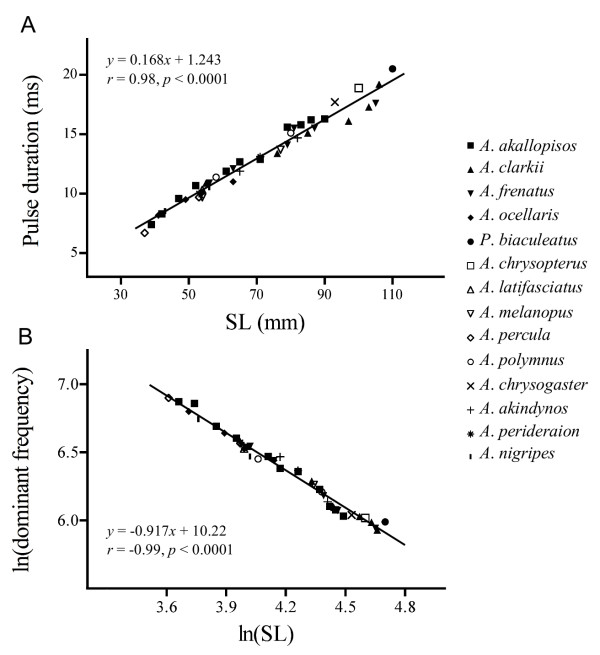
**Influence of fish size (SL) on acoustic variables in 14 clownfish species**. Correlation of (A) pulse duration and (B) dominant frequency against SL. Note that data related to dominant frequency were ln-transformed because they were exponentially related to fish size. Fishes ranged from 37 to 110 mm (*n *= 43). The significance level was determined at *p *< 0.05. Results are expressed as mean values of 50 recorded pulses for each individual.

Sounds were produced by all the 14 species during aggressive interactions. The call of each species consisted of short pulses emitted alone or in series, and in a relatively narrow band of low frequencies (Table 1). Pulse duration and dominant frequency were highly related to fish size across species (Figure 1). The more fish size increased, the more pulse duration increased (*r *= 0.98, *p *< 0.0001; Figure 1A), and the more dominant frequency decreased (*r *= -0.99, *p *< 0.0001; Figure 1B). To determine whether these size-related acoustic features evolved in a similar way among species, five groups of individuals were analysed (see methods section for more details). The inter-species comparison using fish size as a covariate showed that pulse duration (ANCOVA, test for common slopes: *F*_4,33 _= 1.812, *p *= 0.150) and dominant frequency (ANCOVA, test for common slopes: *F*_4,33 _= 1.753, *p *= 0.162) did not differ among species, with all the 14 species being situated on the same slope. Thereby, variation among species in both acoustic features was clearly explained by size dimorphism between clownfish species.

**Table 1:** Summary of the acoustic variables recorded for *Amphiprion *and *Premnas *species

	Pulse duration (ms)	Dominant frequency (Hz)	Pulse period (ms)	Number of pulses per train
	
Species (*n*)	mean ± S.D.	mean ± S.D.	mean ± S.D.	mean ± S.D.
*A. percula *(2)	8.2 ± 1.9	853 ± 152	88.8 ± 18.3	2.5 ± 0.8
*A. nigripes *(2)	9.4 ± 1.4	736 ± 123	124.7 ± 18.1	3.8 ± 1.7
*A. ocellaris *(4)	9.7 ± 1.5	742 ± 124	106.9 ± 21.7	2.2 ± 0.4
*A. latifasciatus *(1)	10.3 ± 0.8	674 ± 102	123.5 ± 18.2	3.0 ± 1.3
*A. akallopisos *(11)	12.5 ± 3.4	645 ± 204	73.8 ± 12.4	3.7 ± 2.3
*A. perideraion *(2)	11.0 ± 1.9	650 ± 86	67.8 ± 18.4	3.2 ± 1.7
*A. melanopus *(2)	11.6 ± 2.2	602 ± 96	90.2 ± 22.0	2.6 ± 0.7
*A. polymnus *(2)	13.3 ± 1.9	564 ± 79	97.6 ± 27.4	2.9 ± 1.6
*A. akindynos *(2)	13.3 ± 1.9	554 ± 106	106.1 ± 15.9	3.2 ± 1.6
*A. frenatus *(6)	14.3 ± 2.5	521 ± 123	106.9 ± 24.7	2.5 ± 0.8
*A. clarkii *(6)	15.4 ± 2.9	477 ± 126	109.1 ± 30.7	3.5 ± 1.8
*A. chrysogaster *(1)	17.7 ± 1.3	420 ± 59	114.0 ± 11.1	2.6 ± 1.3
*A. chrysopterus *(1)	18.9 ± 1.1	411 ± 77	160.9 ± 24.9	3.1 ± 1.3
*P. biaculeatus *(1)	20.5 ± 1.6	399 ± 85	123.1 ± 16.0	3.4 ± 1.7

All recordings were made at 26°C. Species are presented in ascending size order

*n*, number of recorded individuals per species with 50 sounds analysed per individual. Thus, the standard deviation was sometimes calculated from 300 measurements (in case of 6 recorded individuals per species) or from 50 measurements when only one specimen per species was recorded.

**Figure 2:**
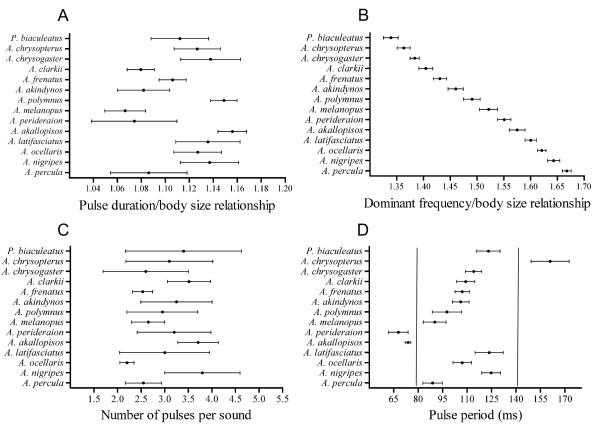
**Variation of acoustic features in 14 clownfish species**. Results are represented as means ± 95% confidence intervals. Vertical lines have been added between sets of overlapping species to indicate gaps.

Some differences between species still remained for the frequency-size and duration-size relationships even after removing the effect of body size (Figures 2A,B). However, this observation needs to be carefully interpreted because deeper attention to pairwise comparisons revealed that pulse duration/body size relationship (*H *= 8.332, d.f. = 4, *p *= 0.0801) and dominant frequency/body size relationship (*H *= 7.276, d.f. = 4, *p *= 0.1220) were not significantly different between individuals having similar body size (53-54 mm SL) and belonging to five different species (*A. latifasciatus, A. melanopus, A. ocellaris, A. percula *and *A. perideraion*). The number of pulses broadly overlapped between species (Figure 2C, Table 1), although there were some differences (*H *= 47.62, d.f. = 13, *p *< 0.01). Pairwise comparisons showed a few species were significantly different (Dunn's test, *p *< 0.01): *A. ocellaris *and *A. nigripes, A. ocellaris *and *A. clarkii, A. ocellaris *and *A. akallopisos*. Pulse period displayed the most variation among species (*H *= 383.1, d.f. = 13, *p *< 0.001), but considerable overlap in pairwise comparisons showed that several species were similar (Dunn's test, *p *> 0.05; Figure 2D, Table 1).

### Morphology and sounds

**Figure 3:**
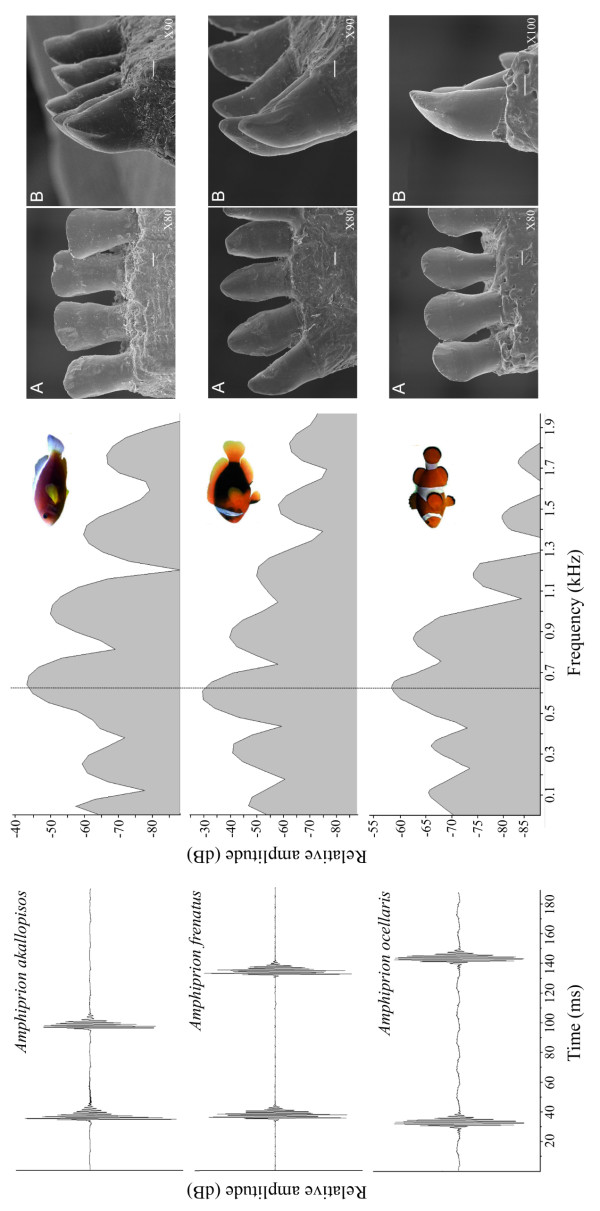
**Oscillogram, power spectrum and SEM pictures of the buccal teeth in *Amphiprion akallopisos, A. frenatus *and *A. ocellaris***. A: ventral view of the teeth from the inner side of the mandible and B: left lateral view of the front teeth of the mandible. Scale bar = 100 μm.

Deeper attention was given to three species having different teeth shape. Sound comparisons of these three species based on three specimens having the same size (61-63 mm SL) revealed that the dominant frequency of *A. akallopisos, A. ocellaris *and *A. frenatus *was not significantly different (*H *= 0.0207, d.f. = 2, *p *= 0.9897) and was respectively 646, 627 and 625 Hz. Moreover, the acoustic waveform and the power spectrum exhibited the same pattern despite the different types of teeth: rectangular and incisiform in *A. akallopisos*, conical and caniniform in *A. frenatus *and spatulate in *A. ocellaris *(Figure 3).

## Discussion

This study is one of the first comparisons of acoustic characteristics in a sizeable number of closely related species of fishes [see also [31]]. The most important insight was found at the level of the relationship between fish size and both dominant frequency and pulse duration. These kinds of relationships were already well known in fishes and have been found in numerous species from different taxa [26,27,32-36]. In the present study and for the first time, it appears that these relationships are observed at a higher taxonomic level in the case of clownfishes (*i.e*. spread over the entire tribe Amphiprionini; see [18]) since dominant frequency and pulse duration are strongly predicted by body size among the 14 different species. It clearly shows all clownfish species use the same mechanism of vocalization [30], which has remained largely conserved throughout evolution. Moreover, different species having different body shape and different types of teeth (*A. akallopisos, A. frenatus *and *A. ocellaris*) but the same size produce sounds that display the same power spectrum and the same oscillogram, demonstrating these morphological variations do not make significant differences in the sounds produced by the jaw teeth snapping. The size influence highlights that all the fourteen species might have a major overlap at the level of pulse length and dominant frequency. Surprisingly, using the pulse duration/body size and dominant frequency/body size ratios removes the effect of body size at the level of pulse duration (Figure 2A), but not at the level of dominant frequency (Figure 2B). Regarding the dominant frequency, the ratio continues to be smaller in larger species, but highly reduces the overlap between species. For example, some *Amphiprion clarkii*, measuring between 55 and 110 mm in SL, produced a frequency range that was between 700 and 400 Hz, overlapping also the frequency of smaller *A. ocellaris *(625-900 Hz; see also [27]). However, the ratio of dominant frequency/body size was 1.40 in *A. clarkii *and 1.62 in *A. ocellaris*, clearly distinguishing the species. Therefore, a factor other than body length must be important in determining the absolute frequency values. The volume of the swimbladder could be the determining factor. Moreover, the data of this last factor are also correlated with fish size. The question arises whether the fish is able to determine the relationship (or the discrepancy) between the emitter size and the sound frequency it detects. If so, it could enable the fish to distinguish whether the emitter is conspecific or not. In the future, it would be interesting to compare these size-related relationships between *Amphiprion *and other pomacentrids. Comparison with *Pomacentrus partitus *data [36] seems to indicate these fish might also be placed on the same slope. In this case, we could expect there is an ancestral relationship between dominant frequency and body size, and this relationship is not important in the taxon diversification because it does not change between species. However, more precise data from other pomacentrids are needed.

Variations in sounds are usually considered as pre-zygotic isolating mechanisms leading to speciation [7,37,38]. In *Amphiprion *species, acoustic properties can contribute to the differentiation of species because some of them showed differences in at least one of the acoustic characteristics recorded. However, the significant overlap in acoustic data due in part to the conserved mechanism of sound production supports the acoustic communication cannot be considered as the unique isolating barrier and does not seem to be the main driving force in the evolution of clownfishes. The fact that all species have the same biomechanics implies these fishes do not have many possibilities to develop variations in their calls: they can differ in dominant frequency and pulse duration through their body size, in pulse period and in number of pulses in a call. The alternative hypothesis would be some differences in the calls are a by-product of the size variation. Body size as a trait of natural selection has already been demonstrated in the speciation event of some stickleback species in which this difference is thought to be an adaptation to alternative foraging habitats [39]. In damselfishes, evolutionary change in body size (*i.e*. gigantism or nanism) could be assumed as a relatively common phenomenon [40,41] and could therefore be involved in the isolation of some *Amphiprion *species. From the proximal cause point of view, the size appears really important in maintaining the hierarchy existing within groups. In *Amphiprion akallopisos*, all aggressive interactions (biting, chasing, frontal and lateral display, body jerking) appear to be preferentially directed towards individuals adjacent in ranks [22]. In *Amphiprion percula*, rank was the only factor associated with the probability of mortality; low-rank individuals suffer from a higher mortality rate than high-rank individuals. The most likely explanation for this pattern is competition for rank [21], preventing smaller fish from having access to reproduction. It means that acoustic communication can be an important factor for mating access.

From the ultimate cause point of view, competition for the limited anemone resource may have resulted in niche partitioning through specialization for different anemone species [42], most clownfish species remaining in close contact with their hosts and rarely interact with other species on the reef [17]. However, some clownfish species appear to partition the anemone resource with other species by having a refuge in size [42,43]: small *A. sandaracinos *or *A. leucokranos *cohabit with *A. chrysopterus *in the region of Madang (Papua New Guinea), while small *A. perideraion *use the same host sea anemone as *A. clarkii *in the region of Okinawa (Japan). In both cases, the different sizes of cohabiting species imply they possess clear differences in their acoustic repertoire (Table 1), size-related call characteristics such as main frequency and pulse length being a by-product of the evolutionary trait. In the Japanese heterospecific groups, small *A. perideraion *are not considered as competitors and should receive less aggressive attention from larger congener *A. clarkii*. Although *A. clarkii *suppresses the growth and reproduction of *A. perideraion *[44], subadult *A. perideraion *are able to mature in heterospecific groups, and change to female when they are the largest among conspecific members. This suggests that *A. perideraion *in heterospecific groups prepare for reproduction before the disappearance or emigration of larger *A. clarkii*. Thus, they adopt a mating strategy that involves waiting for vacated breeding posts because of their low mobility and a low host density [43].

Due to the relative simplicity of many central and peripheral vocal mechanisms, fish typically lack the ability to produce complex and dynamic, frequency-modulated calls [45]. Vocal differences among fish species are usually due to variations in temporal patterning [27,31]. Pulse period has been shown to be the most important acoustic feature involved in species recognition in pomacentrids [46,47]. Divergence in this character seems to be sufficient to drive pre-zygotic isolation [7], because differences in the calling characteristics are able to prevent the signaller to be considered as a competitor. Myrberg *et al*. [48] conducted playback experiments to test the responsiveness of different *Stegastes *species. Although sounds of each species were able to elicit responses of all the other species, males significantly more responded to sounds of their own species than to sounds from congeners. Interestingly, species that cohabit individual sea anemones (*i.e. A. sandaracinos *with *A. chrysopterus *in the region of Madang, or *A. perideraion *with *A. clarkii *in the region of Okinawa) present a completely different pulse period. As previously stated, non-overlap in this character may have been important in the taxon diversification. However, pulse period is not systematically significantly different among sympatric species: *A. clarkii, A. frenatus *and *A. ocellaris *have the same pulse period range (Figure 2D) while living in sympatry on the fringing reef around Sesoko island [49]. These three species inhabit different host species, being *Heteractis crispa *for *A. clarkii, Entacmaea quadricolor *for *A. frenatus *and *Stichodactyla gigantea *for *A. ocellaris *[44,49], which suggests overlap in pulse period among these species is of minor importance.

## Conclusion

We predicted that no-overlap in different acoustic features would drive the taxon diversification. However, results surprisingly showed significant overlap in some acoustic features (dominant frequencies and pulse durations) in *Amphiprion *species. It is the first case for which so many different species can be placed on the same slope, giving the opportunity to use the dominant frequency and pulse duration for assessing fish size. This set of observations highlights 1) the use of a highly conservative mechanism, 2) the important role of body size in clownfish ways of life and 3) that this character is not important in this taxon diversification because all the clownfish species maintained the same relationships between fish size and both dominant frequency and pulse duration. However, in some case, the refuge in size could be a way to access to diversification. Significant overlap in sonic features could also be due to the fact that sounds are not produced to find mate, but to defend mate access, which restricts the constraints of diversification. We conclude that sounds do not appear to be the main driving force in the diversification of clownfishes. However, differences in the pulse period between cohabiting species showed that, in some case, sounds can help to differentiate the species, to prevent competition between cohabiting species and to promote the diversification of taxa.

## Methods

### Sound recording and analysis

Forty-three specimens belonging to 14 species were audio-recorded, and 50 sounds per individual were analysed. Different methods were used to collect acoustic data. On the one hand, sounds were recorded during fieldwork in the lagoons in front of Toliara (Mozambique Channel, west coast of Madagascar, 23°36'S - 43°66'E), in front of Opunohu Bay (Moorea, French Polynesia, 17°29'S - 149°51'W) and on a fringing reef in front of Sesoko Station (Okinawa, Japan, 26°39'N - 127°57'E). Fishes were collected by scuba diving and were placed with their anemone host in glass tanks filled with running seawater at a constant temperature of 26°C. On the other hand, recordings were also made on fishes maintained in tanks (T = 26°C) in the Aquarium of La Rochelle (France) and in Oceanopolis (France). Whatever the method used, all recordings were performed in standard aquaria. Sound recordings and analyses were carried out according to the methodology used by Parmentier et al. [25] and Colleye et al. [26]. The following sonic features were measured: pulse duration (ms), number of pulses in a train, pulse period (ms) and dominant frequency (Hz). Other variables were removed because they are not independent: 1) the interpulse interval (measured as the time from the end of one pulse to the beginning of the next one) was correlated with the pulse period and 2) the sound duration depended on the number of pulses in a call.

### Character reconstruction of acoustic signals

Acoustic characters were obtained from recordings of living fishes. Some acoustic characteristics, such as dominant frequency and pulse duration, vary in a predictable fashion with the size of the calling individual [26,27]. Because differences in these features between species might simply reflect an effect of differences in body size, the size-frequency and size-duration relationships were taken into account rather than the variables alone. Ideally, these relationships should be determined within each species, but we had limited sample size for some species used in the analyses. Accordingly, all individuals' means were used as data points in a linear regression analysis, and the overall significant slope was used to remove the effects of body size from the among-species comparison. Note that data related to dominant frequency were first ln-transformed because they were exponentially related to fish size. Then, the among-species comparison was made by pooling together individuals belonging to the same species.

### Coding of acoustic characters

In this study, we focused on variation in agonistic sounds between closely related species. Usually, phylogenetic studies deal with behavioural characters and score the presence or absence of a given display. However, variation in most acoustic characters is quantitative rather than categorical. Quantitative characters are often used in phylogeny reconstruction but methods of coding these characters continue to be debated [50,51]. Therefore, variation among species for each character was examined according to published protocol [7]. This method is based on the criterion of non-overlap of 95% confidence intervals to define gaps, and divides each acoustic variable into one or more sets of overlapping intervals. Each set was coded as a single character state (see Figure 2 in results). This coding method is relevant because it provides a consistent way of comparing character change across species [7].

### Morphological study

Sound being initiated by teeth collision [see [30]], the buccal dentition of three different species (*A. akallopisos, A. frenatus *and *A. ocellaris*) was studied, with three individuals from each species used to make comparisons among tooth shapes. Fishes were deeply anaesthetised with tricaine methanesulphonate MS-222 in seawater (500 mgl^-1^) and were fixed in 7% buffered seawater formalin for approximately 2 weeks before being transferred to 70% ethanol for storage. Experimental and animal care protocols followed all relevant international guidelines and were approved by the ethics commission (no. 728) of the University of Liège.

After having been removed from the fish, the buccal jaws were minutely cleaned using whet clamps and a little brush. After dehydration, samples were critically point-dried with CO_2 _using a Leica EM CPD030, and platinum coated using a Balzers SCD 030. The material was then examined using a Jeol JSM-840 A scanning electron microscope.

### Statistical analyses

The data used in the analyses were mean values of all recorded sounds for each individual. Because we had limited sample size for some species, an analysis of covariance (ANCOVA) was run to test whether acoustic variables related to fish size evolved in a similar way among five different groups. The first four groups corresponded to species for which we have a sufficient sample size (*A. akallopisos, A. clarkii, A. frenatus *and *A. ocellaris*, see Table 1) while the fifth group was a pool grouping all individuals of the other species with limited sample size (only one or two individuals). The comparison aimed to determine whether the intraspecific size-related variation of the acoustic variables is similar to the interspecific variation (the pool of species). Note that data about dominant frequencies were first ln-transformed because they were exponentially related to fish size. The other two acoustic variables (pulse period and number of pulses per sound) were tested for the assumption of normality (Shapiro-Wilk test), and then they were analyzed using a non-parametric Kruskal-Wallis one-way analysis of variance by ranks with subsequent Dunn's test for pairwise comparisons to test differences between species. Statistical analyses were carried out with Statistica 7.1. Results are presented as means ± S.D. Significance level was determined at *p *< 0.05.

## Acknowledgements

The authors would like to thank J.M. Ouin (Institut Halieutique des Sciences Marines, University of Toliara) and Prof. M. Nakamura (Sesoko Station, Tropical Biosphere Research Center, University of the Ryukyus) for helping to collect fishes and for providing hospitality and laboratory facilities. Many thanks to P. Morinière (Aquarium La Rochelle, France), D. Barthélémy (Océanopolis, France) and C. Michel (Aquarium Dubuisson, Belgium) for allowing free access to fishes during sound recordings. A previous version of the paper benefited greatly from interesting comments made by Dr D. Adriaens (GU) and U. Schliewen (ZSM). We are also greatly indebted to Felix Breden and three anonymous reviewers for their insightful comments and helpful criticism of the original version of the manuscript. OC was supported by a grant from the Belgian National Fund for Scientific Research (Bourse de Doctorat F.R.S.-FNRS). EP is a Research Associate of the F.R.S.-FNRS. This research was supported by the FRFC grants from the F.R.S.-FNRS (no. 2.4.535.10), ANR (ANR-06-JCJC-0012-01), MOM (06 PF 15) and CRISP Program (Coral Reef Initiative in the South Pacific - C2A).

## Authors' contributions

EP designed the study. OC, DL and EP performed sound recordings, OC carried out the experimental work and the sound analysis. EP and OC wrote the paper with input from DL, PV and DL. All authors read and approved the final manuscript.
